# Pediatric primary care and subspecialist providers’ comfort, attitudes and practices screening and referring for social determinants of health

**DOI:** 10.1186/s12913-021-06975-3

**Published:** 2021-09-13

**Authors:** Yonit Lax, Eleanor Bathory, Sandra Braganza

**Affiliations:** 1 General Pediatrics, Population Health, Maimonides Children’s Hospital, SUNY Downstate Medical Center, 1301 57th Street, NY 11218 Brooklyn, USA; 2 Academic General Pediatrics, Social Pediatrics, Children’s Hospital at Montefiore, Albert Einstein College of Medicine, 3544 Jerome Avenue, NY 10467 Bronx, USA

**Keywords:** Social Determinants of Health, Pediatrics, Screening, Low-Income, Pediatrician, Family Medicine

## Abstract

**Background:**

Early detection and management of poverty-related disorders is a recommended pediatric practice; however, little is known about variations of practice between pediatric primary care physicians and subspecialists. The objectives of this study were to assess (1) provider perceptions and attitudes toward caring for low-income children in an urban academic medical center, and (2) variations between primary care physicians and subspecialists in social and financial needs screening and referral practices for low-income children.

**Design/Methods:**

Primary care providers (pediatric and family medicine) and subspecialists providing direct patient care in an urban academic medical center (response rate = 24 %, n = 85/356) completed a 24-item survey (adapted with permission from the AAP Periodic Survey of Fellows No.90) assessing feasibility and comfort screening and addressing social and financial needs, rates of screening for financial hardship, and referrals to local resources. Chi-square tests were performed.

**Results:**

Among respondents, 88 % (75/85) reported comfort caring for low-income children, while 28 % (24/85) reported comfort inquiring about social and financial needs and 34 % (29/85) referring to community resources. Primary care providers more commonly than subspecialists screened for childcare (80 % vs. 59 %, *p* = 0.04), parental: employment (84 % vs. 59 %, p = 0.01), education (40 % vs. 17 %, *p* = 0.02) and mental health (86 % vs. 46 %, *p* = 0.0001), and less commonly screened for transportation (47 % vs. 73 %, *p* = 0.01). Primary care providers more commonly referred for public health insurance (74 % vs. 39 %, *p* = 0.001), public food assistance (30 % vs. 12 %, *p* = 0.04), and adult mental health services (65 % vs. 44 %, *p* < 0.05).

**Conclusions:**

In an urban academic institution serving a population with high poverty rates, pediatric providers feel comfortable providing medical care for low-income children but lack comfort screening and addressing SDH. Though most feel it is their job to refer to resources, less than half felt it was feasible to screen for or address financial needs. Pediatric primary care providers report higher rates of screening and referring than subspecialists. Understanding variations in practice and perceptions among primary care providers and subspecialists may aid in creating interventions to increase screening and referral rates.

## Background

Adverse social conditions in childhood contribute to an elevated burden of disease throughout the life course.[1–3] Pediatric health care providers interact with families regularly in childhood, affording them a unique opportunity to recognize and address the social and economic needs of families. The early detection and management of poverty-related disorders is an emerging component of the pediatric scope of practice, however little is known regarding current pediatric provider practices.[1] Recent studies suggest a wide range of experiences and practices among pediatricians caring for low-income children.[4, 5].

In recent years, the pediatric community has increasingly recognized the importance of screening for and addressing social determinants of health as a component of practice. The American Academy of Pediatrics (AAP) and the American Academy of Family Physicians (AAFP) now recommend that pediatricians and family practice physicians increase knowledge and understanding of social determinants of health, broadening the responsibilities of the physician to include screening, assessment and referrals for physical, emotional or social problems that adversely affect the health of their patients.[6, 7] Collaboration between members of the health care team, including primary care clinicians and subspecialists, is a critical component of effectively supporting families with their medical, social and economic needs.[7, 8].

The most recent AAP Bright Futures guidelines recommend primary care providers screen and address social determinants of health as part of routine pediatric preventative care.[9] Yet, non-primary care services account for approximately 19 % of pediatric visits,[10] and pediatric subspecialist providers often treat more medically complex children, a population that is known to have a high prevalence of unmet social needs.[11–13] Though there is recent literature reporting efforts to increase SDH screening practices among subspecialists, there are not one set of guidelines across subspecialty groups.[14] Thus, we sought to understand more about the different attitudes and practices screening for social and economic needs among pediatric subspecialist providers and primary care providers.

## Methods

### Setting and Study Population

This study was conducted between September and November of 2016 at the Children’s Hospital at Montefiore, an academic tertiary care facility located in Bronx County, New York. The Children’s Hospital at Montefiore, is a safety net hospital providing care for a largely underserved community. In the Bronx, approximately 41 % of children live below the poverty line,[15] and 38 % of children live in food insecure households.[16].

Surveys were distributed to all 356 Montefiore Pediatric and Family Medicine physicans providing direct patient care, including: 286 pediatric faculty members at The Children’s Hospital at Montefiore, 40 Family Medicine Faculty members at Montefiore Medical Center and 30 Family Medicine or Pediatric physicians in non-teaching roles at the Montefiore Medical Group outpatient practices. None of these practices were routinely or universally screening for social needs at the time of this survey. The locations and populations served varied from site to site, however all providers were caring for patients in the Bronx, New York. Access to social workers or behavioral health support also varied by site. Primary care providers were defined as pediatric or family medicine physicians performing well child visit (n = 120) and subspecialists defined as all other providers (n = 236). Medical trainees, including residents, were excluded from this study. The Albert Einstein University institutions review board approved this study and deemed it exempt.

### Survey

We administered a 24-item survey via email using the SurveyMonkey© online tool (SurveyMonkey.com, LLC, Palo Alto, California) to 356 total providers assessing attitudes and practices regarding caring for low-income children, specifically: (1) comfort addressing social and financial needs (2) attitudes regarding screening for social and financial needs, (3) social and financial needs screening practices, (4) community resource referral practices. Survey questions were adapted with permission from the AAP Periodic Survey of Fellows on “Low Income Children” (No. 90). The AAP Periodic Surveys are conducted by the AAP on topics that support its’ strategic mission 3–4 times per year, surveying a random and nationally representative sample of its membership.[17] The social needs questions in the AAP Periodic Survey of Fellows were based on a tool used in a previous study of pediatric residents and had been previously refined in collaboration with the AAP Poverty and Child Health Leadership Workgroup. [2] For the purposes of this study, questions were further adapted based on pilot testing with pediatricians in our institution and feedback from the IRB that ensured respondent confidentiality and anonymity.

*Comfort addressing social and financial needs* was assessed through 3 questions assessing comfort: (1) providing medical care to low-income children, (2) inquiring about financial and related social needs and, (3) referring families for financial and related social needs. For analysis responses answered on a 4-point Likert scale were dichotomized to not at all and somewhat comfortable compared with moderately and very comfortable.

*Attitudes regarding screening for social and financial needs* were assessed through 3 questions “How strongly do you agree or disagree with the following: (1) It is feasible to screen for family financial and related social needs routinely at health care visits, (2) It is important to screen for family financial and related social needs routinely at health visits and, (3) It is my job to refer parents to available clinic and community resources when financial hardship and related social needs are identified?” Response options were scored on a 5-point Likert scale and for analysis responses were dichotomized as strongly agree and agree compared to neutral, disagree and strongly disagree.

*Social and financial needs screening practices* were assessed by asking, “How often do you routinely ask low-income parents about the following: parent educational status, parent employment status, parent mental health, need for childcare, transportation barriers, food insecurity, housing insecurity, utilities/heating insecurity?” Response options were almost always (≥ 75 % of visits), usually (50-74 % of visits), sometimes (25-49 % of visits), and almost never (< 25 % of visits). For analysis responses were dichotomized to sometimes, usually, and almost always screening compared to never screening.

*Community resource referral practices* were assessed by asking “Within the past 12 months, have you referred a low-income family to any of the following community resources: employment/job search services, adult educational services/job training programs, adult mental health providers, childcare centers/providers, Head Start sites or other preschool programs for early childhood development, transportation assistance, local food pantries/private charities, public food assistance (i.e. WIC, School Lunch, Supplemental Nutrition Assistance Program), public health insurance enrollment assistance (e.g., Medicaid, State Children’s Health Insurance Program), housing services, and utility assistance programs?” Response options were yes and no.

### Statistical Analyses

This is a cross-sectional study that was conducted over a of 3 month period. A bias analysis compared gender, age and race between primary care providers and subspecialist providers. Frequencies among primary care providers and subspecialist providers were calculated for (1) comfort addressing social and financial needs (2) attitudes regarding screening for social and financial needs, (3) social and financial needs screening practices, (4) community resource referral practices.

Unadjusted associations between frequencies among primary care providers were compared to those among subspecialist providers using chi square analysis. Data analyses were performed by using SPSS statistical software (SPSS Inc, Chicago, Illinois), with a 2-tailed p-value < 0.05 considered statistically significant.

## Results

### Demographics

Survey response rate was 24 % (85/356), with a response from 17.4 % (41/236) of subspecialists and 36.7 % (44/120) of primary care providers. Table 1 describes the 85 respondents. The mean age was 48, 70 % were female, and 67 % were white. There were no statistically significant differences in age, sex, or race/ethnicity between respondents in primary care providers compared to subspecialists.

**Table 1:** Characteristics of study population

	Primary care *n* = 44	Subspecialist *n* = 41	All Providers *n* = 85
**White**	30 (68 %)	27 (65 %)	57 (67 %)
**Hispanic/ Latino**	3 (6.8 %)	2 (4.9 %)	5 (5.9 %)
**Black/African American**	6 (13.6 %)	1 (2.4 %)	7 (8.2 %)
**Asian**	5 (11.4 %)	11 (26.8 %)	16 (18.8 %)
**Female**	33 (75 %)	26 (63 %)	59 (69.4 %)

*p value > 0.05, not significant

### Comfort addressing social and financial needs

Table 2 shows that most respondents (88 %, 75/85) in both groups reported comfort in providing care for low-income children, while fewer reported comfort inquiring about (28 %, 24/85) or referring for (34 %, 29/85) social needs. There was no statistically significant difference between primary care providers and subspecialist providers.

**Table 2:** Provider comfort addressing social and financial needs

Moderately/Very Comfortable:	Primary care *n* = 44	Subspecialist *n* = 41	All Providers *n* = 85
Providing medical care to low-income children	40 (91 %)	35 (85 %)	75 (88 %)
Inquiring about financial and social needs	14 (32 %)	10 (24 %)	24 (28 %)
Referring families for financial and social needs	13(36 %)	16 (32 %)	29 (34 %)

*p value > 0.05, not significant

### Attitudes regarding screening for social and financial needs

The majority of respondents in both groups reported they believed it was their job to refer patients to resources for financial hardships (76 %, 65/85) (Table 3). Fewer reported it was feasible to screen for financial needs (42 %, 36/85) or that they were well prepared to address families’ financial or social needs (21 %, 18/85). There was no statistically significant difference between the primary care providers and the subspecialist providers.

**Table 3:** Provider attitudes addressing social and financial needs

**Agree or Strongly Agree:**	**Primary care***n*=44	**Subspecialist***n*=41	**All Providers***n*=85
“It is my job to refer patients to resources for financial hardships”	36 (82%)	29 (70%)	65 (76%)
“It is feasible to screen for financial needs routinely at health care visits”	17 (39%)	19 (46%)	36 (42%)
“I am well prepared to address families’ financial and social needs”	10 (23%)	8 (20%)	18 (21%)

### Social and financial needs screening practices

**Fig. 1:**
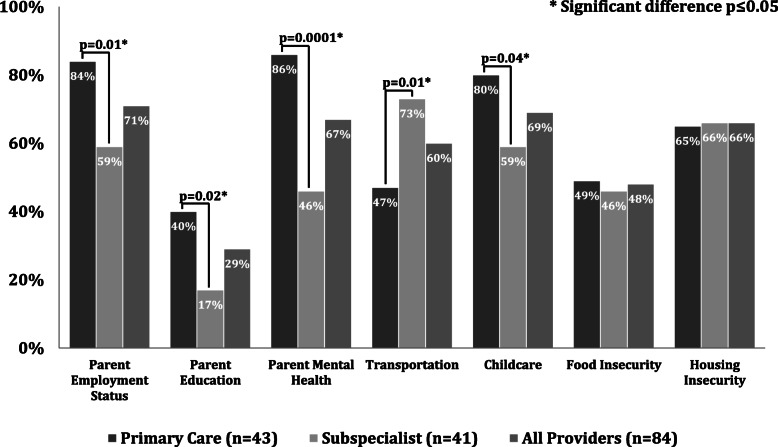
Social and financial needs screening practices

The social needs most commonly screened for were parent employment status, parent mental health, childcare and housing insecurity (Fig. 1). Compared to subspecialist providers, primary care providers reported more commonly screening for parent employment status (84 % vs. 59 %, *p* = 0.01), parent education (40 % vs. 17 %, *p* = 0.02), parent mental health (86 % vs. 46 %, *p* = 0.0001), and childcare (80 % vs. 59 %, *p* = 0.04), and less commonly screening for transportation (47 % vs. 73 %, *p* = 0.01) (Fig. 1).

### Community resource referral practices

**Fig. 2:**
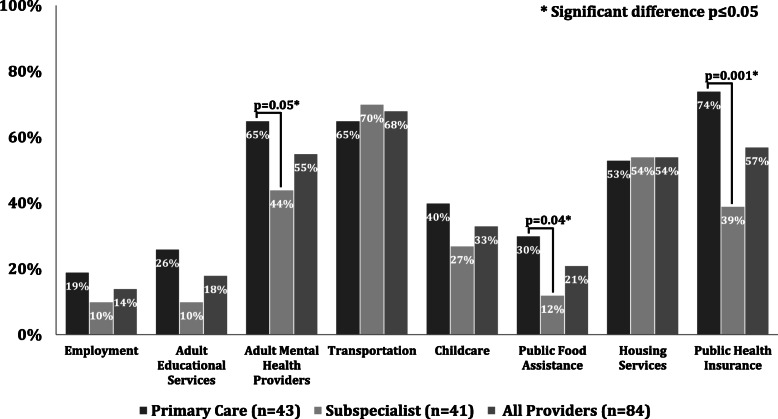
Community resource referral practices

More than half of primary care providers reported referring families for public health insurance, transportation, adult mental health, and housing services, while more than half of subspecialist providers reported referring families for transportation and housing services (Fig. 2). Primary care providers were more likely than subspecialist providers to have reported making community referrals for assistance acquiring public health insurance (74 % vs. 39 %, *p* = 0.001), public food assistance (30 % vs. 12 %, *p* = 0.04), and adult mental health providers (65 % vs. 44 %, *p* < 0.05).

## Discussion

We found that the majority of pediatric providers felt comfortable providing care for low-income children and reported it was their job to refer patients for resources; however, few felt well prepared to do so or believed that it was feasible. This disconnect between the feeling of responsibility to address the social and economic needs of families, and ability to do so is supported by prior studies.[4, 18] Despite recommendations by the largest American professional associations for both pediatricians, the AAP, and family physicians, the AAFP, for physicians to routinely screen for social determinants of health, a minority of providers routinely do.[4, 19, 20] The finding that a majority of providers did not feel well prepared to address financial and social needs suggests they need further training and support. One strategy is to support collaborations between providers and community organizations and local governments, improving pathways connecting patients with community resources that routinely address the financial and social needs identified.

Primary care providers reported higher rates of screening and referrals for several social and economic domains including public health insurance, public food assistance, and adult mental health providers. One influential factor may be the integration of the patient centered medical home model (PCMH) in pediatric primary care clinics which directed primary care physicians to compassionately deliver comprehensive and coordinated care through a family centered approach, in contrast to the more traditional and focused non-primary care facilities.[21] Higher rates of screening and referral for adult mental health services may reflect the 2010 AAP clinical report on postpartum depression that emphasized the rational and need to identify and address postpartum depression in pediatric primary care [22]. Higher rates of screening and referral to for public health insurance among primary care providers may be because patients and families are generally seen in a primary care clinic prior to seeing a subspecialist. Additionally primary care clinics may be less likely to turn patient’s away based on insurance status, but need to screen and refer for public health insurance in order to refer for subspecialist care. Higher rates of screening for public food assistance by primary care providers is supported by prior research showing provider concerns that screening for food insecurity is not an appropriate use of subspecialty evaluations.[23] Additionally, primary care providers may have higher rates of screening and referrals for public food assistance due to the necessary medical documentation they are completing which is required by the WIC program. [24]

Subspecialty providers in this study report significantly higher rates of screening and referring for transportation barriers than primary care providers. Transportation is the second most common social and economic factor screened for by pediatricians in the United States.[4] More than 67 % of US pediatricians report screening for transportation needs, [4] a similar prevalence to the 60 % found in this study. Furthermore, 20 % of children living in urban communities, such as those seen by the providers in this study, have been shown to have transportation barriers to timely health care.[25] Subspecialists often provide care to more medically complex children with chronic diseases, who are more likely to have transportation barriers to hospital discharge and adherence to medical appointments. [26] Chronic disease management requires adequate access to transportation, as patients have more frequent medical visits to clinicians and pharmacies.[27] Additionally, subspecialists often provide care to patients in a wider catchment area than primary care providers, therefore transportation barriers may be a larger issue, accounting for the more frequent screening and referrals.

More than half of pediatric providers studied reported screening and referring patients for housing issues, with no significant difference between subspecialists and primary care providers. Traditionally in pediatrics, the social history includes the number and relationship of people in the household.[28] More recently, housing insecurity and poor housing conditions have been widely acknowledged as a key social determinant of health associated with poor health outcomes, developmental risks, and hospital readmissions in pediatrics. [29–32] Therefore, expanding to ask a question about the household such as housing stability and conditions may be a natural extension for providers.

Our study has several limitations. (1) Due to the nature of being a single center study in an urban area primarily serving low-income children, the findings may not be generalizable. (2) The 24 % response rate was low and a participation bias may have affected our findings, however this may be expected in studies sampling physicians particularly when survey topics are sensitive [33]. (3) There is a shared electronic medical record system that was used by all of the primary care and subspecialist physicians. It is possible that subspecialists had reviewed social and economic needs and interventions noted in the notes of primary doctors and thus did not feel it was necessary to address further. (4) The survey was measured by self-report and therefore may be limited by recall bias and social desirability bias. (5) This study was completed in 2016, and the medical landscape has continued to evolve regarding screening for and addressing social and financial needs in the primary care setting. However, screening for and addressing social and financial needs is still not a commonly reported practice among pediatric subspecialty providers.

## Conclusions

This study found that pediatric primary care providers report higher rates of screening and referring for social determinants of health than subspecialist providers do in most domains. However, both primary care providers and subspecialists report a desire to provide care surrounding social and economic factors but feel limited by the feasibility and inadequate training around implementing this in practice. Understanding the variations in practice, comfort and attitudes between primary care providers and subspecialists may aid in creating meaningful interventions to address the needs of pediatric patients and their families. Teaching trainees about social determinants of health has been shown to increase knowledge and changes to practice,[18, 34] which suggests that appropriate professional development focused on screening and addressing social and economic needs has potential to shift the practice of all pediatric providers. Thus, further research is needed to understand how to effectively and efficiently educate practicing primary care and subspecialty providers, and improve screening and referral.[14].

## Data Availability

the datasets used and analysed during the current study are available from the corresponding author on reasonable request.
